# Optimizing Airway Management Strategies for Anterior Neck Hematoma Following Parotidectomy

**DOI:** 10.7759/cureus.84774

**Published:** 2025-05-25

**Authors:** John A Merlo, Hamed Sadeghipour

**Affiliations:** 1 Anesthesiology and Critical Care, Sisters of St. Mary (SSM) Saint Louis University Hospital, St. Louis, USA

**Keywords:** airway management, difficult airway, intubation, neck hematoma, perioperative medicine

## Abstract

There is minimal research on the true incidence of postoperative neck hematomas following parotidectomy. Managing difficult airways in patients with an anterior neck hematoma requires extreme caution due to the high risk of complete airway collapse. In this case report, a 79-year-old male diagnosed with parotid squamous cell carcinoma underwent a parotidectomy and subsequently developed an anterior neck hematoma as a postoperative complication. Airway management was achieved by maintaining spontaneous ventilation and carefully titrating intravenous and inhaled anesthetics, followed by surgical incision of the hematoma. The use of a video laryngoscope with a D-blade and the initial avoidance of neuromuscular blockade facilitated safe airway control. This case highlights the critical need for prompt intervention in post-parotidectomy neck hematomas and underscores the importance of coordinated efforts between surgical and anesthesia teams. Additionally, strict adherence to established airway management protocols and the application of varied techniques are vital for ensuring positive patient outcomes.

## Introduction

Limited research exists regarding the true incidence of postoperative neck hematoma following parotidectomy, largely due to underreporting in the current literature [1]. Parotidectomy complications include facial nerve injury, Frey’s syndrome, hematoma, salivary fistula, infection, sensory deficits, and cosmetic deformity, with hematoma risk particularly elevated due to the gland’s rich vascularity, proximity to the facial nerve, and potential for intraoperative or postoperative bleeding from transected intraglandular vessels [2]. Bleeding and hematomas after parotidectomy are uncommon and are usually related to inadequate hemostasis at the time of the surgical procedure, with incidences reported between 1.7% and 10.5% [2]. While some patients with neck hematomas are managed conservatively through observation, case reports underscore the challenge in predicting the onset of sudden airway collapse [3]. In this case presentation, we discuss a 79-year-old male diagnosed with parotid squamous cell carcinoma who experienced a postoperative complication of an anterior neck hematoma. Through the implementation of multiple airway techniques and surgical interventions, successful airway control was achieved without any complications.

## Case presentation

A 79-year-old male, with an ASA score of 3 and a past medical history including hypertension, type 2 diabetes mellitus, and right parotid squamous cell carcinoma, underwent a right parotidectomy. During the initial surgery, the right parotid mass encasing the jugular vein and the spinal accessory nerve (cranial nerve XI) and involving the prevertebral fascia was dissected with nerve preservation. The surgery lasted approximately three hours. Four hours later, the bedside nurse noted the development of swelling in the right neck and face, prompting a surgical evaluation and the scheduling of an urgent right neck hematoma evacuation.

**Figure 1:**
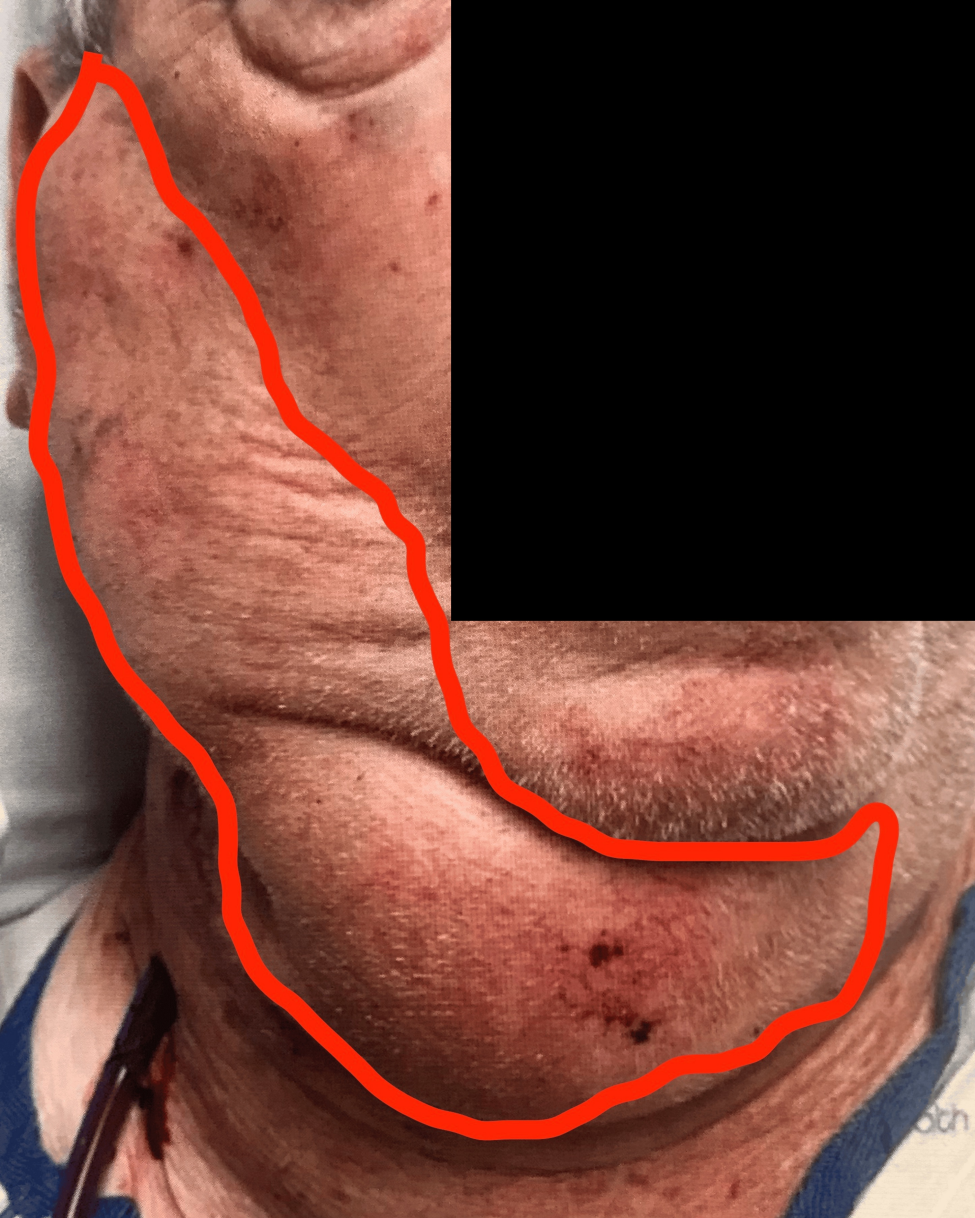
Right-Sided Anterior Neck Hematoma The right anterior hematoma is outlined in red

Upon admission to the preoperative suite, the patient was seated upright. His blood pressure was 160/82 mmHg, heart rate 105 bpm, respiratory rate 16 breaths per minute, and oxygen saturation was 94% on 5 L/min of oxygen via non-rebreather facemask, administered to maintain oxygenation above 92%. The patient was alert, with a Glasgow Coma Scale score of 15. He reported increasing neck and facial swelling but exhibited no stridor or pain.

The anesthesia team was immediately available, and the plan was collaboratively discussed by the attending surgeon, anesthesiologist, and residents. The patient remained NPO as instructed, with status appropriately verified. Once the operating room was ready, he was promptly transported. The operating room was equipped with a video laryngoscope with MAC 4 and D blades, a bougie, fiberoptic scope, intubating laryngeal mask airway, various sizes of endotracheal tubes, and a cricothyroidotomy kit as a precaution. In addition, appropriate medications were prepared. The patient was positioned in a slight upright posture and preoxygenated with mask ventilation. At that time, vital signs included a blood pressure of 137/77 mmHg, heart rate of 110 bpm, and oxygen saturation of 98% on 10 L/min of oxygen via facemask. At this time, an appropriate pause was observed, and the plan was discussed with the entire team in the operating room. It was agreed that, in the event of difficulty with ventilation or endotracheal tube placement, the best course of action would be immediate evacuation of the hematoma. The surgical team acknowledged the plan and was ready at the bedside to proceed if needed. The cricothyroidotomy kit was prepared, and the neck was prepped accordingly.

Due to the patient’s age and recent surgery, midazolam and ketamine were avoided to ensure full cooperation and reduce the risk of postoperative delirium. However, the patient was unable to tolerate a fully awake airway evaluation due to discomfort. To minimize patient distress, the airway and extent of the hematoma were initially assessed while maintaining face mask ventilation with 10 L/min of oxygen, a fresh gas flow of 10 L/min, and 1.0% inspired sevoflurane, with continuous capnography monitoring. Using a video laryngoscope with a MAC 4 blade, the blade was inserted into the patient’s mouth. However, no airway view was obtained, as the patient began to cough suddenly, prompting abortion of the attempt. Face mask ventilation was subsequently resumed. While not compromising oxygenation, 30 mg of propofol and 25 mcg of fentanyl were carefully titrated. At this point, it was evident that the expanding hematoma was obstructing the view, and further manipulation with a fiberoptic scope could exacerbate airway irritation and increase the risk of bleeding.

The face mask remained in place, and assisted ventilation continued with 10 L/min of oxygen, a fresh gas flow of 10 L/min, and 1.6% inspired sevoflurane, with continuous capnography monitoring to facilitate patient relaxation. Despite efforts, a Grade 4 view remained, obstructed by the hematoma. Gentle cricoid pressure was applied while carefully avoiding compression of the hematoma, along with patient repositioning. However, neither intervention was sufficient to improve the view. At this time, a 100 mm oral airway was inserted while maintaining face mask application with assisted ventilation. In collaboration with the surgical team, the decision was made to proceed with surgical evacuation. The surgical team made a vertical incision along the prior surgical incision line. Approximately 20 cc of dark, non-pulsatile blood was evacuated, with clear visualization of hematoma decompression. Before the second attempt, 50 mg of propofol was administered, and sevoflurane was continued. Using a D-blade video laryngoscope, improved visibility revealed a Grade 2b/3 view. While maintaining the view, 100 mg of succinylcholine was administered, facilitating successful insertion of a size 7.0 endotracheal tube. Bilateral breath sounds were confirmed, and appropriate end-tidal CO₂ levels were displayed. The tube was subsequently secured. Throughout the procedure, the patient remained hemodynamically stable. An additional 50 mcg of fentanyl was administered for pain control, and sevoflurane was continued. The hematoma was fully evacuated, and a Jackson-Pratt drain was placed. The total surgical duration was two hours. The hematoma was attributed to venous collaterals located lateral to the previous neck dissection. These collaterals were ligated with sutures, and hemostasis was confirmed on inspection prior to closure. The patient was successfully extubated and transferred to the recovery unit.

In the recovery unit, he remained hemodynamically stable on room air. No further postoperative complications were observed. He was discharged four days later to follow up with the surgical team in the clinic.

## Discussion

Anterior neck hematomas after parotidectomy are typically uncommon, with incidences ranging from 1.7% to 10.5% [2]. One study found that 3.4% of anterior neck hematomas were managed by aspiration and compression with no need for surgical exploration [4]. While some patients with neck hematomas are managed conservatively through observation, case reports underscore the challenge in predicting the onset of sudden airway collapse [3]. In a six-year retrospective review of 1,265 surgical procedures, it was found that 6.5% of parotidectomy cases led to hematoma complications requiring surgical exploration [5]. Studies have shown that neck hematomas typically arise from venous or capillary sources rather than arterial bleeding. Yet regardless of their origin, they can insidiously progress to airway obstruction [3]. In our case, it was noted that the neck hematoma originated from a venous source, which was controlled after neck exploration.

Airway management in the setting of an anterior neck hematoma is critically challenging due to rapid airway compression and anatomical distortion. Prompt recognition and timely intervention are essential, as delays can result in complete obstruction and increased morbidity [3]. In hemodynamically stable patients, awake fiberoptic intubation is often preferred, allowing for spontaneous ventilation and visualization of distorted airway structures [6]. Conversely, patients with severe compromise or rapidly expanding hematomas may require emergent surgical airway access, such as cricothyrotomy or tracheostomy [7]. A meta-analysis on anterior neck hematomas following carotid endarterectomy suggests that stable patients can be managed with spontaneous ventilation and awake fiberoptic intubation [8]. The study noted that when fiberoptic intubation failed due to poor patient tolerance, bleeding risk, or instability, direct laryngoscopy, either awake or post-induction, combined with surgical incision decompression was successful [8]. In addition, a case report of neck hematoma following anterior cervical spine surgery described failed awake intubation using both video and fiberoptic techniques due to pharyngeal edema and poor airway visualization. Despite hematoma evacuation via a local incision between the carotid sheath and midline viscera, intubation remained unsuccessful, necessitating an emergent open tracheostomy to secure the airway [9]. Given the unpredictable nature of presentation, multidisciplinary coordination and readiness for surgical airway are vital to ensure safe and effective management.

Moreover, airway impairment may result from soft tissue swelling compressing the trachea. Thus, if feasible, awake intubation with maintenance of spontaneous breathing is favored [6]. In our case, we performed a modified awake intubation, initially using sevoflurane followed by the addition of propofol and fentanyl. This approach was selected to minimize coughing while preserving effective patient communication and spontaneous ventilation. Midazolam was avoided due to the patient’s age, and ketamine was excluded to maintain the patient’s ability to effectively communicate. Furthermore, both medications were avoided to maintain spontaneous ventilation and cooperation while reducing the risk of postoperative delirium. Intubation was performed with careful attention to airway security, prioritizing optimal visualization before administering any neuromuscular blockade.

In a meta-analysis of eight articles encompassing 429 patients with anticipated difficult airways, awake tracheal intubation via video laryngoscopy was compared to fiberoptic bronchoscopy, demonstrating shorter intubation times and comparable first-attempt success rates and patient satisfaction levels [10]. Additionally, in cases of poor direct visualization of the glottis, decompression of the airway via surgical incision facilitated successful tracheal intubation [6].

To our knowledge, this is the first case report to provide a detailed airway management approach for a neck hematoma following a parotidectomy. A systematic review and meta-analysis on postoperative neck hematomas after thyroidectomy identified key risk factors, such as male sex, older age, Graves’ disease, hypertension, antithrombotic use, and extensive surgery, emphasizing the need for vigilant monitoring in high-risk patients [11]. Although focused on thyroidectomy, these findings highlight the broader risk of hematoma across head and neck surgeries. Given the parotid gland’s vascularity and proximity to vital structures, further research is warranted to better define hematoma risk and guide postoperative management in parotidectomy patients.

## Conclusions

In conclusion, we report a case in which surgical incision of the anterior hematoma, coupled with the use of a video laryngoscope with a D-blade, ensured successful airway establishment. In our case, the patient was spontaneously breathing, and neuromuscular blockade was initially withheld due to the uncertainty of securing the airway prior to complete evacuation of the hematoma. This case highlights the urgent need for timely intervention in the management of postoperative neck hematomas following parotidectomy. Furthermore, effective collaboration between the surgical and anesthesia teams played a pivotal role in achieving a favorable outcome.
